# Adolescent Bullying Involvement and Psychosocial Aspects of Family and School Life: A Cross-Sectional Study from Guangdong Province in China

**DOI:** 10.1371/journal.pone.0038619

**Published:** 2012-07-18

**Authors:** Hui Wang, Xiaolan Zhou, Ciyong Lu, Jie Wu, Xueqing Deng, Lingyao Hong, Xue Gao, Yuan He

**Affiliations:** Department of Medical Statistics and Epidemiology, School of Public Health, Sun Yat-sen University, Guangzhou, China; Instituto de Higiene e Medicina Tropical, Portugal

## Abstract

**Background:**

School bullying is an emerging problem in China. The present study aimed to measure the prevalence of bullying behaviors among Chinese adolescents and to examine the association of bullying and being bullied with family factors, school factors and indicators of psychosocial adjustment.

**Methods:**

A cross-sectional study was conducted. A total of 8,342 middle school students were surveyed in four cities in the Guangdong Province. Self-reports on bullying involvement and information regarding family factors, school factors and psychosocial adjustment were collected. Descriptive statistics and multi-level logistic regression analysis were used to evaluate the prevalence of school bullying and explore potentially influential factors.

**Results:**

Of the total sample, 20.83% (1,738) reported being involved in bullying behaviors. Of the respondents, 18.99% were victims of bullying, 8.60% were bullies and 6.74% both bullied themselves and bullied others. Factors that were determined to be correlated with bullying behaviors included grade, parental caring, consideration of suicide, running away from home, time spent online per day and being in a physical fight.

**Conclusion:**

Bullying was determined to be prevalent among Chinese adolescents. Given the concurrent psychosocial adjustment, family and school factors associated with bullying, as well as the potential long-term negative outcomes for these youth, this issue merits serious attention, both for future research and preventive intervention.

## Introduction

Since Olweus published the book “Aggression in the Schools” in 1993, there has been a growing interest in the area of school bullying. The book stated that “a student is being bullied or victimized when he or she is exposed repeatedly and over time to negative action on the part of one or more students ” [1]and that bullying was characterized by an imbalance of power, aggressive behaviors and repetition over time. Data from the recent large-scale Health Behavior in School-aged Children survey (HSBC) conducted among 40 countries suggested that the prevalence of bullying (bullying others, being bullied, and being both a bully and a victim) may range from 8.6% to 45.2% among boys, with a median of 23.4%, and 4.8% to 35.8% among girls, with a median of 15.8% [2]. Another cross-national study, the Global School-based Student Health Survey (GSHS) carried out among middle school students in 19 low- or middle-income countries showed that the prevalence of bullying in countries ranged from 7.8% in Tajikistan to 60.9% in Zambia [3].

Adolescence is a period of immense behavioral, psychological and social changes and challenges [4]. Previous research has indicated that both bullies and victims have an increased rate of submissive and withdrawing behavior. Victims have shown more peer relational difficulties than have uninvolved in bullying participants [5], and they were more likely to have behavioral problems such as substance use, weapon carrying, and even school shootings [5], [6]. There is also increasing evidence suggesting that exposure to violent behavior during childhood can affect individuals into their adulthood and that bullying involvement can act as a precursor to both physical and psychological problems [7]. In Bond’s two year cohort study, a history of victimization among school-aged students was a strong predictor for the onset of self-reported symptoms of anxiety or depression. Being victimized has a significant impact on future emotional well-being, especially for girls [6].

Given the long-term consequences of bullying, there is an urgent need to address this universal problem and to increase the understanding of the larger proximal development mechanisms that may promote or inhibit school bulling. From a review of the literature, we found that the following variables had been identified to be associated with school bullying: 1) Demographic characteristics: Previous studies have indicated that male students report involvement in significantly larger numbers of violent incidents than female students [8], [9]. Additionally, a number of studies have indicated that school bullying declines with increasing age, whereby the younger the students were, the more likely they were to report frequent victimization [10], [11].

2) Family factors: It has been reported that children involved in bullying were more likely to have problems with poor family functioning and an insecure attachment with their parents [12], [13]. Adolescents who lived in intact families and either reported higher involvement in schools or communicated with parents often were less likely to be engaged in bullying [14], [15]. Lower parental support was also reported to be an important predictor for school bullying [16]. In addition, students who lived in a conflictive family environment were also reported to be more likely to bully others than those who have harmonious family relations [17]. In a study by Chen, however, in which student’s pocket money was used as an indicator of Family SES (socioeconomic status), the results did not show any association between family SES and school bullying, which was attributed to the equal family income distribution in Taiwan [8].

3) School factors: The school environment is important for understanding the origins of bully/victim problems and for seeking further avenues for change and prevention [9]. A number of studies have found that poor classmate relations predicted a high level of aggressive behaviors [10]. Teachers play a crucial role in children's wellbeing and development. Care and support from teachers can reduce the aggression and delinquency of their students. In a study by Wei and colleagues, the researchers showed that less support and more maltreatment by a teacher were factors likely to result in higher levels of engagement in adolescent bullying [11]. Other previous studies have indicated that victims showed decreased rates of academic success, measured by lower grades, compared with those not involved in bullying [12], [13]. Glew hypothesized that bullying impaired concentration and subsequent academic achievement in victims [14]. Conversely, in a study by Woods, high academic achievement was an important predictor for relational bullying [15].

4) Psychosocial adjustment: Recently, many researchers have identified the association between psychosocial factors and school bullying. For example, Brunstein found that students who have a history of bullying or being bullied have a higher risk of committing suicide [18]. Those who often felt lonely were more likely to report being a target or aggressor of bullying [19]. In a study by Haynie, it was concluded that children who were involved in bullying were more likely to run away from home [20]. Students who engaged in physical fighting also showed a higher probability for involvement in school bullying [20].

Although we concluded that bullying is a universal phenomenon, it is clear that there are cultural variations in its prevalence and the way that bullying or victimization relates to other factors. Most previous studies, however, have been carried out in Western or developed countries, and only a handful of studies have been conducted in low- or middle-income countries. There is also a paucity of studies on family status (parental communication, family economic status), school dynamics (classmate relations, student-teacher relations) and the personal psychosocial adjustment of students (feeling lonely, attempting suicide) in the Chinese cultural context. Therefore, we carried out this large-scale cross-sectional study among middle school students in the Southern Province of China. The two main purposes of our research were:

1) To examine the prevalence of school bullying, including bullying others, being bullied and being a bully-victim. We have adopted the increasingly common term bully-victim to indicate those students who participate in both bullying and victimization. In view of its dual involvement in bullying and victimization, this emerging group may experience a higher level of psychosocial risks or life events than either bullies or victims.

2) To explore the factors that may contribute to the occurrence of school bullying. The variables highlighted in this study were highly correlated with students’ everyday lives, which is consistent with what has been widely reported from previous research.

## Methods

### Study Design and Participants

A cross-sectional study was conducted to investigate the prevalence of school bullying and to examine the relationship between potentially influential factors and involvement in school bullying. Participants were middle school students recruited from four cities in the Guangdong Province (Guangzhou, Shenzhen, Chaozhou, and Dongguan). Guangzhou and Chaozhou represent the traditional Yue culture. Shenzhen and Dongguan, however, are known as immigrant cities, with more than half of the population migrating from other provinces. The schools in Guangdong were divided into three categories, based on teaching quality: key senior high school, regular senior high school and vocational school. A stratified cluster, random sampling method was used to randomly select participants among the three types of schools. First, two key senior high schools, two regular senior high schools and two vocational schools were selected in Guangzhou, Shenzhen, and Chaozhou; in Dongguan, however, one regular senior high school and two regular junior high schools were selected. Next, two classes were randomly selected from each grade in these schools. All students (a total of 8,342) in the selected classes were invited to participate in this study and provided usable information. The participation rate was 99.7%, and those who asked for sick leave were not included in the study.

### Data Collection

To protect the privacy of the students, anonymous questionnaires were administered by trained interviewers in the absence of the teachers (to avoid any potential information bias). Students were required to fill out the questionnaires during class time. All data were collected between 2009 and 2011.

### Ethical Statement

The study received approval from the Sun Yat-Sen University, School of Public Health Institutional Review Board. Participants were fully informed of the purpose of the study and were invited to participate voluntarily. Written consent letters were obtained from the school, each participating student and either of the student’s parents.

### Measures

#### Independent variables

Socio-demographic variables: Age, grade, gender, student’s pocket money (Students were asked how much pocket money on average they received per month from their parents. The rating choices for this item were 1) lower than 100 Yuan, 2) 100–199 Yuan, and 3) 200 Yuan or more.

Social friends: “Do you have friends who have dropped/are dropping out of school?”.

Family factors: Living arrangement, family economic status, family communications, parental caring. Living arrangement was assessed by asking who lived in the student’s primary home. Family economic status was measured by asking the student’s perception of their family’s current economic status (rated from good to bad). Family communication was assessed by asking the student how often they communicate with their parents on the issues of everyday life (coded on a 3-point scale from often to scarce). Parental caring was assessed by asking, “Are you satisfied with the care or love you receive from your parents, based on a 3-point scale from satisfaction to dissatisfaction?”.

School factors: Classmate relations and teacher-classmate relations were also assessed based on the student’s self-rating about their relationship with classmates and teachers, from good to bad. Academic achievements were captured by a single item asking for a personal appraisal of students’ performances relative to that of their classmates (responses were coded as “above average,” “average,” and “below average”).

Psychosocial adjustment: Feeling lonely was assessed by asking, “During the past 12 months, how often did you feel lonely per week?” Response options ranged from 1 (never) to 4 (over 4 days). Suicide attempts were assessed by asking, “During the past 12 months, did you ever seriously consider attempting suicide?” Responses were categorized into 4 groups: Never, Considered, Planned, and Attempted. Running away from home was assessed by asking, “During the past 12 months, did you run away from home without your parents’ permission for more than 24 hours?” Response options were 1) never, 2) considered, 3) attempted, or 4) have run away from home one time or more. Physical fighting was assessed by asking, “During the past 12 months, how many times did you fight with others?” Time spent online per day was assessed by asking, “How much time do you spend online per day?”.

#### Dependent variables

The questions about bullying and victimization consisted of 12 parts, with the answers given on a 3-point scale as follows: 1-never, 2-sometimes or rarely (one or two times) or 3-often (more than three times).

Bullying and victimization were assessed with parallel questions: “During the last year have you ever been (a1) “hit, kicked, pushed, shoved around, or locked another student indoors?”; (b1) “made fun of or insulted?”; (c1) “excluded intentionally or prevented from participating?”; (d1) “made fun of with sexual jokes, comments or gestures?”; (e1) “blackmailed for money?” or (f1) “bullied in some other way?”.

Question for bullying were as follows: Have you ever (a2) “hit, kicked, pushed, shoved around, or locked another student indoors?” (b2) “made fun of, or teased him or her in a hurtful way?” (c2) “excluded another student intentionally, or prevented another student from participating?” (d2) “made fun of with sexual jokes, comments or gestures to another students?” (e2) “blackmailed money from other students?” (f2) “bullied other students in some other way?”.

Students reporting at least one bullying behavior with a frequency of “often” in the past year were classified as bullies [21]. Victims were those who reported at least one victimization experience in the past year with a frequency of “often.” Bully-victims met the criteria for being both a bully and victim. All other students were labeled as non-bullies/non-victims and served as the comparison group.

### Statistical Analysis

All statistical analyses were conducted using SAS 9.1. Descriptive analyses were used to describe demographic characteristics and the prevalence of school bullying. All factors that were statistically significance in the univariate analysis and that have been widely reported in the literature were further analyzed by multivariate analysis. In the multivariate analysis, a student’s grade, rather than age, was adjusted for in the total sample because grade was a strong predictor for adolescent bullying. Three multi-level logistic regression models were fitted, one for each type of involvement in school bullying. Adjusted odds ratios (OR) were obtained with 95% confidence intervals (CI). Because individuals were grouped into schools, and therefore not independent, a multi-level analysis was carried out to select possible factors that may influence school bullying. The GLIMMIX procedure in SAS was used to fit the two-level logistic regression mixed models in which schools were treated as clusters.

## Results

### Demographic Information

Table 1 and Table 2 provides basic demographic information for the sample. The final sample included 8,342 middle-school students: 4196 boys (50.3%) and 4146 girls (49.7%). The students ranged in age from 10 to 22 years old, and the mean age was 16.4 (±1.63). Overall, 20.83% of the total participants reported being involved in school bullying during the past 12 months, with 18.99% of the students reporting being bullied and 8.6% admitting to bullying others. A subset of students (6.74%) was involved in both victimization and bullying. A total of 27.84% (2322) were from junior high schools and 72.16% (6020) were from senior high schools. A total of 65.39% (5455) students lived with both biological parents, whereas 24.51% (2045) lived in single-parent families. Regarding academic achievement, 5961 (71.46%) students appraised themselves as average and 1361 (16.32%) as below average. A total of 4277 (51.27%) students reported poor relations with classmates, and 36.98% of the participants had poor relations with their teachers. Regarding the psychosocial factors, 0.79% (66) of the students had attempted suicide, 15.5% (1293) felt lonely over 4 days in a week and 1.87% of the total sample had run away from home more than once.

**Table 1:** Demographic Information for the Total Sample and Prevalence of Bullying and Victimization by Demographic group.

Covariate	Total		Bully		Victim		Bully-Victim*	
	N	Percent (%)	N	Percent (%)	N	Percent (%)	N	Percent (%)
Total	8,342	100	717	8.6	1,584	18.99	563	6.71
Grade								
Junior	2,322	27.84	160	6.89	415	17.87	124	5.34
Senior	6,020	72.16	557	9.25	1169	19.42	439	7.29
Gender								
Boy	4,196	50.3	364	8.67	786	18.73	282	6.72
Girl	4,146	49.7	353	8.51	798	19.25	281	6.78
Social (No vs. Yes)								
Yes	4,574	54.83	379	8.29	875	19.13	298	6.52
No	3,616	43.35	324	8.96	685	18.94	254	7.02
Pocket money (RMB)								
<100	4,011	73.1	283	7.06	765	19.07	229	5.71
100–199	3,331	10.15	301	9.04	593	17.80	231	6.93
≥200	939	16.32	126	13.42	214	22.79	99	10.54
Living arrangement								
Two biological parents	5,455	65.56	461	8.45	984	18.04	365	6.69
Only father or mother	2,045	24.58	185	9.05	425	20.78	139	6.80
Others	820	9.86	69	8.41	173	21.10	57	6.95
Parental caring								
Satisfaction	5,970	71.57	431	7.22	1,001	16.77	338	5.66
Average	1,924	23.06	208	10.81	442	22.97	163	8.47
Dissatisfaction	441	5.29	77	17.46	139	31.52	61	13.83
Communication with parents								
Often	3,475	41.66	244	7.02	561	16.14	197	5.67
General	3,478	41.69	308	8.86	662	19.03	243	6.99
Rare	1,375	16.48	162	11.78	359	26.11	121	8.80
Economic status								
Above average	1,987	23.82	170	8.56	357	17.97	131	6.59
Average	4,823	57.82	393	8.15	869	18.02	306	6.34
Below average	1,500	17.98	153	10.20	351	23.40	125	8.33
Academic achievement								
Above average	984	11.8	100	10.16	244	24.80	91	9.25
Average	5,961	71.46	472	7.92	1040	17.45	358	6.01
Below average	1,361	16.32	135	9.92	286	21.01	105	7.71

* bully-victim: Those students who participate in both bullying and victimization.

**Table 2:** Demographic Information for the Total Sample and Prevalence of Bullying and Victimization by Demographic group (Continued).

Covariate	Total		Bully		Victim		Bully-Victim*	
	N	Percent (%)	N	Percent (%)	N	Percent (%)	N	Percent (%)
Classmate relations								
Good	2,175	26.07	166	7.63	358	16.46	129	5.93
Average	1,929	23.12	179	9.28	430	22.29	144	7.47
Poor	4,277	51.27	371	8.67	793	18.54	290	6.78
Relationship with teachers								
Good	1,575	18.88	117	7.43	262	16.63	91	5.78
Average	3,665	43.93	316	8.62	740	20.19	250	6.82
Poor	3,085	36.98	284	9.21	582	18.87	222	7.20
Feel lonely								
More than 4 Days/Week	1,293	15.5	127	9.82	277	21.42	101	7.81
1 to 4 Days/Week	2,551	30.58	239	9.37	507	19.87	174	6.82
Less than 1 Day/Week	1,968	23.59	117	5.95	287	14.58	91	4.62
Never	2,464	29.54	220	8.93	496	20.13	185	7.51
Suicide								
Never	6,591	79.01	495	7.51	1086	16.48	387	5.87
Considered	1,429	17.13	167	11.69	383	26.80	134	9.38
Planned	171	2.05	30	17.54	75	43.86	24	14.04
Attempted	66	0.79	18	27.27	26	39.39	13	19.70
Running away from home								
Never	5,791	69.42	408	7.05	929	16.04	314	5.42
Considered	2,204	26.42	237	10.75	541	24.55	192	8.71
Attempted	178	2.13	38	21.35	60	33.71	28	15.73
Run away one time or more	131	1.57	31	23.66	49	37.40	26	19.85
Fighting								
Never	7,179	86.06	515	7.17	1247	17.37	404	5.63
Once	587	7.04	73	12.44	146	24.87	61	10.39
2–3 times	311	3.73	52	16.72	100	32.15	43	13.83
4 times or more	201	2.41	72	35.82	81	40.30	51	25.37
Time online per day								
Never	880	10.55	32	3.64	143	16.25	27	3.07
<2 h	3,679	44.10	225	6.12	611	16.61	180	4.89
2 h–4 h	2,646	31.72	291	11.00	520	19.65	225	8.50
>4 h	1,073	12.86	165	15.38	303	28.24	130	12.12

* bully-victim: Those students who participate in both bullying and victimization.

### Univariate Analysis for Bully, Victim and Bully-victim Groups

As shown in Table 3 and Table 4, without adjustment for other variables, bully, victim and bully-victim groups were correlated with pocket money, parental caring, communication with parents, feeling lonely, suicide attempts or ideation, running away from home, being in a physical fight and time spent online. Economic status was only significantly correlated with being a victim or bully-victim. There were no significant differences between gender and bully, victim and bully-victim.

**Table 3:** Unadjusted OR (95%CI) for Bullying, Victimization and Bully-victim by univariate analysis.

Covariates	Bully		Victim		Bully-Victim*	
	Unadjusted OR	95%CI	Unadjusted OR	95%CI	Unadjusted OR	95%CI
Gender (Girl vs. Boy)	0.99	0.84–1.15	1.04	0.93–1.16	1.02	0.86–1.21
Grade (Junior vs. Senior)	0.73	0.60–0.88#	0.92	0.81–1.04	0.72	0.59–0.89#
Social friends (No vs. Yes)	0.92	0.79–1.08	1.03	0.92–1.15	1.08	0.90–1.29
Pocket money (RMB)						
<100	1.00		1.00		1.00	
100–199	1.29	1.09–1.53#	0.90	0.80–1.02	1.21	1.00–1.46
≥200	2.03	1.62–2.54#	1.24	1.05–1.48#	1.94	1.51–2.48#
Living arrangement						
Two biological parents	1.00		1.00		1.00	
Only father or mother	1.06	0.89–1.27	1.18	1.04–1.34#	1.00	0.81–1.22
Others	1.00	0.77–1.30	1.22	1.01–1.46#	1.04	0.78–1.39
Parental caring						
Satisfaction	1.00		1.00		1.00	
General	1.57	1.32–1.87#	1.50	1.32–1.70#	1.55	1.28–1.89#
Dissatisfaction	2.69	2.06–3.51#	2.28	1.84–2.82#	2.65	1.98–3.55#
Communication with parents						
Often	1.00		1.00		1.00	
General	1.30	1.09–1.55#	1.24	1.09–1.40#	1.27	1.04–1.54#
Scare	1.81	1.47–2.24#	1.88	1.61–2.18#	1.64	1.30–2.08#
Economic status						
Good	1.00		1.00		1.00	
General	0.96	0.80–1.16	1.02	0.89–1.16	1.02	0.82–1.27
Bad	1.22	0.97–1.53	1.40	1.19–1.65#	1.43	1.11–1.85#
Academic achievement						
Above average	1.00		1.00		1.00	
Average	0.63	0.63–1.00	0.65	0.56–0.77#	0.65	0.51–0.83#
Below average	0.70	0.70–1.22	0.78	0.64–0.95#	0.78	0.58–1.05
Classmate relations						
Good	1.00		1.00		1.00	
Average	1.21	0.97–1.51	1.44	1.23–1.68#	1.21	0.97–1.51
Bad	1.12	0.92–1.36	1.14	0.99–1.31	1.12	0.91–1.39

* bully-victim: Those students who participate in both bullying and victimization.

#:*P*<0.05.

**Table 4:** Unadjusted OR (95%CI) for Bullying, Victimization and Bully-victim by univariate analysis (Continued).

Covariates	Bully		Victim		Bully-Victim*	
	Unadjusted OR	95%CI	Unadjusted OR	95%CI	Unadjusted OR	95%CI
Relationship with teachers						
Good	1.00		1.00		1.00	
Average	1.16	0.93–1.45	1.26	1.08–1.47#	1.18	0.92–1.51
Bad	1.22	0.97–1.53	1.13	0.96–1.33	1.22	0.94–1.57
Feeling Lonely (days/week)						
More than 4 Days/Week	1.00		1.00		1.00	
1 to 4 Days/Week	1.01	0.80–1.28	0.96	0.81–1.14	0.94	0.72–1.22
Less than 1 Day/Week	0.62	0.47–0.82#	0.66	0.55–0.81#	0.63	0.46–0.86#
Never	0.98	0.77–1.25	0.99	0.83–1.18	1.06	0.81–1.39
Suicide attempt or ideation						
Never	1.00		1.00		1.00	
Considered	1.65	1.37–1.99#	1.88	1.64–2.15#	1.68	1.37–2.06#
Planned	2.66	1.77–3.99#	4.00	2.94–5.46#	2.65	1.70–4.13#
Attempted	4.50	2.59–7.82#	3.27	1.99–5.40#	3.82	2.06–7.09#
Running away from home						
Never	1.00		1.00		1.00	
Considered	1.61	1.36–1.90#	1.72	1.52–1.94#	1.68	1.39–2.03#
Attempted	3.52	2.42–5.12#	2.63	1.91–3.62#	3.18	2.09–4.84#
Running away one time or more	4.06	2.67–6.17#	3.14	2.18–4.51#	4.30	2.75–6.72#
Fighting						
Never	1.00		1.00		1.00	
Once	2.67	1.95–3.66#	1.60	1.31–1.95#	1.60	1.31–1.95#
2–3 times	7.44	5.48–10.11#	2.31	1.80–2.96#	2.31	1.80–2.96#
4 times or more	2.00	1.51–2.67#	3.29	2.46–4.40#	3.29	2.46–4.40#
Time online per day						
Never	1.00		1.00		1.00	
<2 h	1.73	1.18–2.52#	1.02	0.84–1.25	1.62	1.08–2.45#
2 h–4 h	3.22	2.21–4.68#	1.23	1.00–1.51	2.88	1.91–4.33#
>4 h	4.85	3.28–7.17#	2.01	1.61–2.52#	4.37	2.85–6.68#

* bully-victim: Those students who participate in both bullying and victimization.

#:*P*<0.05.

### Multilevel Logistic Regression Analysis: Bully

The final logistic regression model for bullying is presented in Table 5 and Table 6. Six of the original variables remained in the final model: grade, parental caring, considered suicide, running away from home, being in a physical fight and time spent online per day. Students who were dissatisfied with parental caring were 1.7 times more likely to be bullies than those who were satisfied with parental caring. Students who spend more time online were also at a higher risk of being bullies. Finally, students who had considered suicide (OR = 1.26, 95% CI = 1.02–1.56) or attempted to run away from home (OR = 1.89, 95% CI = 1.22–2.83) had a higher probability of being bullies.

**Table 5:** Adjusted OR (95% CI ) for Bullying, Victimization and Bully-victim by multi-level logistic regression.

Covariates	Bully		Victim		Bully-Victim*	
	Adjusted OR	95%CI	Adjusted OR	95%CI	Adjusted OR	95%CI
Gender (Girl vs. Boy)	1.04	0.88–1.22	1.06	0.94–1.20	1.07	0.89–1.29
Grade (Junior vs. Senior)	0.36	0.27–0.48#	0.57	0.45–0.72#	0.26	0.18–0.36#
Pocket money (RMB)						
<100	1.00		1.00		1.00	
100–199	1.09	0.90–1.30	0.84	0.73–0.96	1.07	0.87–1.31
≥200	1.31	1.00–1.69	1.01	0.83–1.23	1.27	0.96–1.69
Living arrangement						
Two biological parents	-	-	1.00		-	-
Only father or mother	-	-	1.04	0.83–1.29	-	-
Others	-	-	1.09	0.94–1.26	-	-
Parental care						
Satisfaction	1.00		1.00		1.00	
General	1.30	1.06–1.59#	1.09	0.95–1.25	1.35	1.08–1.69#
Dissatisfaction	1.70	1.24–2.32#	1.28	1.07–1.53#	1.85	1.31–2.59#
Communication with parents						
Often	1.00		1.00		1.00	
General	1.11	0.92–1.34	1.16	1.00–1.35	1.04	0.84–1.29
Scare	1.11	0.86–1.42	1.55	1.22–1.98#	0.99	0.75–1.31
Economic status						
Good	-	-	1.00		1.00	
General	-	-	1.02	0.88–1.18	1.10	0.87–1.38
Bad	-	-	1.28	1.06–1.55#	1.48	1.11–1.98#
Academic achievement						
Above average	-	-	1.00		1.00	
Average	-	-	0.55	0.43–0.69#	0.45	0.31–0.65#
Below average	-	-	0.39	0.16–1.00	0.16	0.02–1.24
Classmate relations						
Good	-	-	1.00			
Average	-	-	1.44	1.17–1.77#	-	-
Bad	-	-	1.31	1.03–1.66#	-	-

* bully-victim: Those students who participate in both bullying and victimization.

#:*P*<0.05.

“-”: Factors not included in the final multivariate analysis.

**Table 6:** Adjusted OR (95% CI ) for Bullying, Victimization and Bully-victim by multi-level logistic regression (Continued).

Covariates	Bully		Victim		Bully-Victim*	
	Adjusted OR	95%CI	Adjusted OR	95%CI	Adjusted OR	95%CI
Relationship with teachers						
Good	-	-	1.00			
Average	-	-	1.00	0.83–1.21	-	-
Bad	-	-	1.02	0.82–1.28	-	-
Feeling Lonely (days/week)						
More than 4 Days/Week	1.00		1.00		1.00	
1 to 4 Days/Week	1.15	0.90–1.48	0.64	0.26–1.59	0.34	0.05–2.58
Less than 1 Day/Week	0.85	0.63–1.16	0.50	0.20–1.24	0.29	0.04–2.19
Never	1.18	0.85–1.64	0.63	0.25–1.60	0.42	0.05–3.19
Suicide attempt or ideation						
Never	1.00		1.00		1.00	
Considered	1.26	1.02–1.56#	1.51	1.29–1.76#	1.28	1.02–1.62#
Planned	1.27	0.79–2.03	2.45	1.73–3.49#	1.23	0.74–2.06
Attempted	1.49	0.73–3.01	1.46	0.80–2.66	1.06	0.48–2.37
Running away from home						
Never	1.00		1.00		1.00	
Considered	1.24	1.02–1.51#	1.28	1.11–1.48#	1.29	1.04–1.61#
Attempted	1.89	1.23–2.88#	1.62	1.14–2.31#	1.86	1.17–2.98#
Running away one time or more	1.47	0.86–2.51	1.33	0.86–2.06	1.72	0.97–3.03
Fighting						
Never	1.00		1.00		1.00	
Once	2.11	1.59–2.81#	1.64	1.32–2.03#	2.30	1.69–3.12#
2–3 times	2.95	2.09–4.17#	2.34	1.78–3.08#	3.14	2.16–4.57#
4 times or more	6.56	4.57–9.41#	2.52	1.80–3.53#	4.78	3.21–7.12#
Time online per day						
Never	1.00		1.00		1.00	
<2 h	1.86	1.21–2.74#	1.07	0.87–1.33	1.91	1.22–2.99#
2 h–4 h	3.10	2.01–4.53#	1.19	0.95–1.48	2.98	1.91–4.67#
>4 h	4.37	2.68–6.36#	1.85	1.43–2.38#	4.28	2.65–6.89#

* bully-victim: Those students who participate in both bullying and victimization.

#:*P*<0.05.

“-“: Factors not included in the final multivariate analysis.

### Multilevel Logistic Regression Analysis: Victim

The final model for victimization in Table 5 and Table 6 showed many correlations. Good economic status appeared to protect students from being bullied. Students who were dissatisfied with their parental caring or scarcely communicated with their parents were at a higher risk to be bullied. Adolescents who reported that they had attempted to run away from home were 62% more likely to be bullied. Those who spent more than 4 hours/day online also had a higher probability to be bullied (OR = 1.85, 95% CI = 1.43–2.38).

### Multilevel Logistic Regression Analysis: Bully-victim

Eight independent variables out of the original factors remained in the final model. With these reference categories, it was determined that senior students were less likely to be in the bully-victim group. Students who were dissatisfied with their parental caring, scarcely communicated with their parents, had attempted suicide or tried to run away from home, however, were more likely to be bullied and to bully others.

## Discussion

In this study, we found that school bullying was not rare in China, and many risk factors for bullying exist throughout school and family life. Similar studies have already been reported. A cross-sectional self-report survey of 11–15 year-old school children in 27 countries revealed substantial cross-national differences, with a low prevalence of involvement in bullying in Sweden (14.6% and 15.4% of children reporting victimization and bullying, respectively) and a high prevalence in Lithuania (56.3% and 54.9% of children reporting victimization and bullying) [22]. In one of the few studies of school bullying in China, Chen reported that 68% of the middle school students studied had been bullied at least once during the previous year [8]. A possible explanation for the variance in prevalence could be differences in study design and the nature of the samples. In addition, socioeconomic diversity and various cultural definitions and understandings of bullying behaviors may have contributed to the variance of the bullying rate [23], [24], [25].

Contrary to our expectation, we found that senior students reported a higher level of both bullying and being bullied than junior students, which was consistent with the findings in a study by O'Connell, notably, that older boys were more likely to actively bully than were younger boys [26]. Mrug also reported that higher levels of aggressive fantasies, delinquency and overt aggression were well-predicted by older age [27]. Previous studies also suggested that boys were more likely to report being a target or aggressor of bullying than girls [17], [28], whereas in the present study, there was no significant gender difference. This finding is consistent with some previous studies 12,15,29] arguing that the rate of bullying among boys and girls may be similar. The difference may be reflected in the forms of bullying. Boys usually practice physical and direct bullying (e.g. kicking someone), while girls may engage in psychological bullying (i.e. spreading rumors) [30], [31].

Family function can also contribute to the establishment and maintenance of a bully-victim relationship. Previous research suggested that both avoidant attachment and preoccupied attachment have been found to predict aggressive behaviors and victimization concurrently and over time [31]. Our research further supported the notion that the students who were dissatisfied with their parental care were more likely to engage in bullying or be a victim of bullying. It is also worth noting that good communication with parents reduced the probability of victimization as well. Through communication, students confide their problems to their parents and seek better ways to handle them. A study by Reese suggested that a lack of emotional support was negatively correlated with school bullying [32]. The results of the present study showed that good economic status may act as a protective factor for being bullied, which was consistent with a study by Qiao [12]. The same relations that were previously noted among bullies, however, were not identified here. A study by Kim suggested that students with a high family socioeconomic status were more likely to become persecutors [33]. Thus, further study is needed to explore the different impacts that family factors have on bullying and victimization.

Victimized adolescents may be widely disliked or not well-liked by their peers, and adolescents were likely to seek out targets who have been isolated by their peer group without receiving any negative evaluation [23]. Victimized adolescents often experience peer rejection and deviant affiliation, leaving them more vulnerable to aggressive peers. Asian children are, perhaps, more likely to be influenced by their peers and to mimic their behaviors than non-Asian children [8]. In our results, victims tended to have poor relationships with their classmates. Bullies were also disliked amongst classmates but were less socially isolated than victims, primarily due to their popularity with other aggressive and deviant adolescents [24]. Contrary to our expectations, students with poor relationships with teachers did not show a higher probability of school bullying and victimization. When confronted with peer abuse or peer rejection, adolescents mostly turned to their parents or peers rather than teachers for help [25], which may suggest that emotional support from peers and parents was more important than that from teachers to these students.

Our results also indicated that victimization was significantly correlated with suicidal attempts. A prior study has already indicated that the peer rejection and abuse inherent in school bullying may have a direct effect on the genesis of suicidal ideation. We presumed that the correlations may be caused by the same influential factors shared by both suicide attempts and victimization. For instance, lack of social support and deviant peer associations are consistently and highly-correlated with school bullying exposure and are significantly associated with suicide attempts [13]. Running away from home occurred equally among those who were bullied and those who were bullies, which was consistent with a study by Haynie [18]. Some have hypothesized that running away from home may be used as an adaptation to a stressful structural circumstance [18], [34]. Contrary to our expectation, students who had run away from home or tried to commit suicide did not display a higher risk for school bullying. We presumed that the smaller sample of this category may have caused the false negative. Additionally, due to the nature of cross-sectional studies, a causal relationship cannot be determined. Students who have tried to commit suicide or run away from home more than once may have gotten more attention from both parents and teachers, preventing them from engaging in future risky behaviors.

Our results clearly showed an association between bullying and being bullied and engaging in a physical fight. Liang has already reported that experiencing bullying puts younger adolescents at a higher risk for physical fighting [35], which reiterates the importance of addressing these serious public health issues among adolescents. Another interesting finding in our study was that students who spent more time on the internet had a higher risk of engaging in bullying or being a victim of bullying. One possible explanation is the increased access to various formats of violence and bullying. Adolescent problem behaviors should be seen as socially-learned adaptations in a multi-level ecological context. The internet has provided adolescents with convenient access to a culture of violence. In addition, young people are socially connected with others through the internet, and it has become a new medium for bullying behavior [36]. The direction of the link between bullying behaviors and internet use, however, is difficult to determine due to the nature of this cross-sectional study; they might mutually reinforce each other, thereby formulating a vicious circle.

The nature of the sample of students in this study needs to be considered when interpreting these results. We reported results from data among adolescents in Guangdong, and the findings may only be representative of the adolescents in this area, and not the rest of the country. The findings in the present study are based on classroom samples and will not be representative of adolescents outside of school settings, who may be at the highest risk for involvement in bullying. The data were based only on self-reporting, so the possibility of biased reporting motivated by a desire to provide socially desirable responses must be recognized. Recall bias may have also affected the reporting of violent behavior, which spanned a 12-month period prior to the survey.

As a random sampling method was used with a cross-sectional design, the sample in this study appears to represent the population well; however, its limitations must also be considered. First, being a cross-sectional study, causal inferences regarding relational factors and involvement in bullying cannot be made. Thus, while it appears that victims of bullying are more likely to internalize problems, it is also possible that students who internalize their problems are more likely to be targeted by bullies. Longitudinal designs should be utilized to address this shortcoming. Future research should focus on a broader spectrum of predictors over time to identify causal determinants of violence in this population. Second, self-reported data may lead to under- or over-reporting. Future studies should collect information from multiple sources, such as teachers or parents. Thirdly, the study did not provide information regarding all potential or likely risk factors, such as self-esteem and violent behavior of friends or parents.

This paper provides some information about the prevalence of bullying and being bullied and the possible influential factors associated with bullying and being bullied. With regard to future research, programs may benefit from studies that include more nuanced measures of context, especially family- and school-related factors (e.g., parental monitoring, sibling relationships, class size and gender ratio). Furthermore, research examining mediator models could illuminate the mechanisms by which the psychosocial risk factors studied herein may give rise to involvement in bullying and victimization.

### Conclusion

In conclusion, this study investigated the prevalence of school bullying among Chinese middle school students by utilizing a large-scale survey sample. It also further examined the effects of potentially influential factors on adolescent bullying and victimization. We found that 20.83% of the participants were involved in school bullying, and a series of factors were proven to be significantly correlated with school bullying. Previous research has indicated that school bullying can be prevented. Due to the frequency of bullying episodes, schools are the target of the most intervention efforts. The prevalence of bullying observed in Chinese middle school students in this study suggests the importance of preventive intervention research to target bullying behaviors. Effective preventive measures require full consideration of the social and environmental factors that would inhibit bullying behaviors among Chinese adolescents. School-wide interventions, such as the Olweus Bullying Prevention Program (BPP), have been recognized to be some of the most effective strategies for bullying behaviors. The BPP utilizes a multi-pronged approach, incorporating school-wide (e.g., formation of a bullying prevention coordinating committee), classroom-level (e.g., class meetings with parents) and individual activities (e.g., direct interventions with identified bullies, victims and their parents) [29]. In light of the extent of school bullying, and its contribution to the development of other youth problems, concerted efforts to implement preventive measures are necessary.
